# Validation Thin Layer Chromatography for the Determination of Acetaminophen in Tablets and Comparison with a Pharmacopeial Method

**DOI:** 10.1155/2013/545703

**Published:** 2013-08-25

**Authors:** Alina Pyka, Marika Budzisz, Małgorzata Dołowy

**Affiliations:** Department of Analytical Chemistry, Faculty of Pharmacy, Medical University of Silesia, PL-4 Jagiellońska Street, 41-200 Sosnowiec, Poland

## Abstract

Adsorption thin layer chromatography (NP-TLC) with densitometry has been established for the identification and the quantification of acetaminophen in three leading commercial products of pharmaceutical tablets coded as brand: P1 (*Product* no. 1), P2 (*Product* no. 2), and P3 (*Product* no. 3). Applied chromatographic conditions have separated acetaminophen from its related substances, namely, 4-aminophenol and and 4′-chloroacetanilide. UV densitometry was performed in absorbance mode at 248 nm. The presented method was validated by specificity, range, linearity, accuracy, precision, detection limit, quantitative limit, and robustness. The TLC-densitometric method was also compared with a pharmacopeial UV-spectrophotometric method for the assay of acetaminophen, and the results confirmed statistically that the NP-TLC-densitometric method can be used as a substitute method. It could be said that the validated NP-TLC-densitometric method is suitable for the routine analysis of acetaminophen in quantity control laboratories.

## 1. Introduction

Acetaminophen (paracetamol) has pharmacological and pharmaceutical significance. It is a nonsteroidal anti-inflammatory drug and is used for the reduction of pain and fever. Acetaminophen is commonly used for the relief of headaches and other minor aches and is a major ingredient in numerous cold and flu remedies.

Acetaminophen was quantitatively determined in different biological fluids, namely, plasma [1–6], urine [7–12], serum [11, 13], and tissue [14]. Moreover, acetaminophen was also determined in different pharmaceutical preparations in single and in combined dosage forms [15–30]. 

As was reported in the literature, several methods like liquid chromatography [1, 4, 5, 21], high performance liquid chromatography [3, 8, 10, 12, 17, 18], reversed-phase sequential injection chromatography (SIC) [20], spectrophotometric [7, 9, 13, 16], spectrophotometric with spectrodensitometric [22], spectrofluorimetric [15], capillary electrophoresis [11, 19], colorimetric [2, 6], chronoamperometric [14], thin layer chromatography with spectrophotometric [23], and thin layer chromatography with a fluorescence plate reader [24], and thin layer chromatography (HPTLC) with a densitometry were successfully applied in qualitative and quantitative acetaminophen analysis [25–30]. Generally, HPLC and UV-spectrophotometric methods have been reported in the United States and Polish Pharmacopeias for the analysis of acetaminophen in pharmaceutical preparations [31, 32].

Dimitrovska et al. [23] described the conditions for the determination of propyphenazone, acetaminophen, caffeine, and codeine phosphate in commercial tablet dosage with preparative thin layer chromatography. The separation of propyphenazone, acetaminophen, and caffeine was performed by use of a mobile phase chloroform + acetone + ammonium hydroxide (25%) in volume rations 8 : 2 : 0.1. Codeine phosphate was separated from the other components with chloroform + ethanol in volume ration 8 : 2, as a mobile phase. UV-spectrophotometric determinations of propyphenazone, acetaminophen, caffeine, and codeine after their separation on thin layer and elution from the adsorbent were performed. Tavallali et al. [24] developed a method to determine caffeine and acetaminophen concentrations in pharmaceutical formulations using TLC with a fluorescence plate reader. Separation of acetaminophen and caffeine was performed using the mobile phase n-hexane + ethyl acetate + ethanol (2.5 : 1.5 : 0.4, v/v).

Many papers described the determination of acetaminophen in single and in combined tablet dosage forms using TLC with densitometry [24–30]. However, authors did not separate the related substances (4-aminophenol and 4′-choroacetanilide) to acetaminophen. This fact indicates that the previous TLC-densitometric methods presented in cited previous papers were not validated in term of specificity accordance to validation guidelines [33, 34]. Ferenczi-Fodor and coworkers described in their papers that besides the typical validation characteristics such as accuracy, precision, repeatability, specificity, detection limit, quantification limit, linearity, and range, in the case of liquid chromatography the evaluation of robustness should be considered [34, 35]. It is a critical parameter having significant effect for final results of chromatographic analysis. It was stated that when this parameter is not constant, the analytical process should be improved [34, 35]. According to ICH guideline, the robustness test includes the influence of the variations of the following parameters like variations of pH in a mobile phase, variations in mobile phase composition, different column and temperature on final results of liquid chromatographic analysis [33].

Presented work is a continuation of our previous TLC acetaminophen study. In earlier paper we described the preliminary investigations for the analysis of acetaminophen by TLC with densitometry including the choice of the optimal chromatographic conditions enabled to complete separation of examined acetaminophen from its related substances (4-aminophenol and 4′-choroacetanilide) such as silica gel 60F_254_ plates and a mixture of chloroform + acetone + ammonia (25%) in volume compositions 8 : 2 : 0.1 as a mobile phase [36].

The aims of this work were to

- elaborate the conditions for quantitative determination of acetaminophen in tablets by a TLC-densitometric method with regard to obligatory validation presented in validation guidelines and in Ferenczi-Fodor and also Nagy-Turák reports including robustness test [33–35];
- apply the spectrophotometric method recommended by Polish Pharmacopoeia to the quantitative determination of acetaminophen in tablets [31];
- compare the results of quantitative determination of acetaminophen in tablets obtained by TLC-densitometric and spectrophotometric methods.

Based on the obtained results the usefulness of the validated TLC-densitometric method for quantitative analysis of acetaminophen in combined dosage form in comparison to spectrophotometric method (recommended by Polish Pharmacopoeia) was estimated. Moreover, the use of the changes of the following chromatographic conditions such as sorbent type, the chamber type, extraction time, the temperature of plate activation, the distance of development, the wavelength, and the analyst as the new factors in robustness test of TLC-densitometric method was discussed.

## 2. Materials and Methods

### 2.1. Apparatus

Densitometer: Camag (Muttenz, Switzerland) equipped with TLC Scanner.

Spectrophotometer: Specord 205 (Analytik Jena, Germany).

IKA Ultra-Turrax Tube Drive Workstation with BMT-20-S Tube for grinding with balls of stainless steel.

NP-TLC plates: 10 × 20 cm glass plates precoated with 0.20 mm layers of silica gel 60F_254_ (E.Merck, #1.05570); 20 × 20 cm aluminium plates precoated with 0.20 mm layers of silica gel 60F_254_ (E.Merck, #1.05554).

The 5 *μ*L Camag micropipettes were used to apply the solutions to the plates.

Chromatographic chambers: twin-trough chamber for 20 × 20 cm plates (#0.222.5255, Camag, Muttenz, Switzerland) and twin-trough chamber for 20 × 10 cm plates (#0.222.5221, Camag, Muttenz, Switzerland).

### 2.2. Chemicals

Acetaminophen (Sigma-Aldrich, St. Louis, MO, USA), testing and handling conforms to United States Pharmacopeia, 4-aminophenol (>99%, Sigma-Aldrich, St. Louis, MO, USA), and 4′-chloroacetanilide (>98%, Sigma-Aldrich, St. Louis, MO, USA), were used as standards. All chemicals and reagents used for TLC were analytical grade and were purchased from POCh, Gliwice, Poland.

### 2.3. Pharmaceutical Preparation

Pharmaceutical preparations of three different pharmaceutical manufactures containing 500 mg of acetaminophen per tablet (*Product 1*) and also 500 mg per dragged tablet (*Product 2* and *Product 3*) were investigated.

### 2.4. Preparation Sample of Tablets

Ten tablets were ground for 20 min with a speed equal to 4000 rpm using an IKA Ultra-Turrax Tube Drive Workstation with a BMT-20-S tube for grinding with balls of stainless steel. The obtained powders of acetaminophen tablets (equivalent to 25 mg acetaminophen by weighing the powder to an accuracy of 0.1 mg) were shaken with ethanol (99.8%, 10 mL) for 5 min with a speed equal to 4000 rpm. After shaking, the solutions were filtered through a medium-density filter to volumetric flasks (25 mL) and replenished using ethanol (99.8%) to demanded volume. From these solutions, were next prepared the solutions with about the concentrations of active substances (acetaminophen) equal to 0.30 mg · mL^−1^, 0.20 mg · mL^−1^, and 0.10 mg · mL^−1^. These solutions (5 *μ*L) were used for the TLC-densitometric analysis and for quantitative determination of acetaminophen in certain pharmaceutical preparations. The equivalent of 100 mg acetaminophen by weight of the powder was also used for UV-spectrophotometric analysis.

### 2.5. Preparation of Standard Solution of Acetaminophen and Its Related Substances

Standard solutions of acetaminophen and standard solutions of 4-aminophenol and 4′-chloroacetanilide were prepared by dissolving the solutes in ethanol (99.8%).

### 2.6. Thin Layer Chromatography

The plates were prewashed with methanol and dried for 24 h at room temperature. Before use the plates used in NP-TLC were activated at 120°C for 10 min. TLC-densitometric method for determination of acetaminophen was performed on aluminium plates precoated with 0.20 mm layers of silica gel 60F_254_ (E.Merck, # 1.05570). Additionally, 20 × 20 cm aluminum plates precoated with 0.20 mm layers of silica gel 60F_254_ (E.Merck, # 1.05554) were used to test robustness. The robustness of an analytical procedure is a measure of its capacity to remain unaffected by small but deliberate variations in method parameters. 

The solutions of acetaminophen samples and acetaminophen standards (5 *μ*L) were spotted manually on the chromatographic plates. 

The mixture of chloroform + acetone + ammonia (25%) in volume compositions 8 : 2 : 0.1 was used as mobile phase. Of the mobile phase, 50 mL was used in all cases. After saturation of the twin-trough chamber (20 cm × 20 cm) with the mobile-phase vapor for 15 min, the plates were developed vertically at room temperature (20°C) to a distance of 7.5 cm. The plates were then dried for 20 h at room temperature (20°C) in a fume cupboard.

Additionally, a twin-trough chamber of 20 × 10 cm (#0.222.5221, Camag) was used to test robustness.

### 2.7. Densitometric and Spectrodensitometric Investigations

Densitometric and spectrodensitometric investigations were done using a TLC Scanner 3 operated in the absorbance mode. The radiation source was a deuterium lamp emitting a continuous UV spectrum between 190 and 450 nm. Densitometric scanning was then performed at multiwavelength in the range of 200 to 400 nm, at wavelength intervals of 50 nm at each step. Finally, densitometric scanning, for quantitative determination of acetaminophen, was then performed at absorption maximum of acetaminophen equal to 248 nm. The chromatographic bands obtained on the densitograms were also investigated by spectrodensitometric analysis under the following conditions. The slit dimensions were 10.00 × 0.40 mm, Macro; the optimal optical system was light; the scanning speeds were 20 mm · s^−1^ and 20 nm · s^−1^, respectively, for densitometric and spectrodensitometric analysis; the data resolution was 100 *μ*m · step^−1^ and 1 nm · step^−1^, respectively, for densitometric and spectrodensitometric analysis; the measurement type was remission; and the measurement mode was absorption.

### 2.8. Validation of the TLC Method

The proposed method was validated by specificity, range, linearity, accuracy, precision, detection limit, quantitative limit, and robustness according to the ICH guidelines [33] and according to the guidelines described by Ferenczi-Fodor et al. [34].

#### 2.8.1. Specificity

The specificity of the method was checked by chromatography of working standard (acetaminophen) and related substances (4-aminophenol and 4′-chloroacetanilide) and sample solution of acetaminophen extracted from tablets.

#### 2.8.2. Linearity of Detector Response and Range

The linearity of the TLC method was evaluated by analysis of nine standard solutions of acetaminophen of concentrations 0.08, 0.10, 0.12, 0.15, 0.20, 0.25, 0.30, 0.35, and 0.40 mg · mL^−1^. The solutions (5 *μ*L) were applied to the same plate. The plates were developed using above-mentioned mobile phases (in thin layer chromatography section) and scanned. The experiments were performed in six different analyses.

#### 2.8.3. Accuracy

The accuracy of the TLC method was evaluated by measurement of recovery. Known amounts of acetaminophen standards in the low, medium, and high levels of the calibration plot were added to powdered tablets of known acetaminophen content, and the tablets were extracted and analyzed under the optimized conditions. The experiments were performed in six different analyses.

#### 2.8.4. Precision

Repeatability (intraday precision) of the method was determined by analysis of three replicates of three sample solutions (ethanol extracts of acetaminophen) of different concentrations (0.10, 0.20, and 0.30 mg · mL^−1^) under the same operating conditions over a short interval of time (the same day). Intermediate (interday) precision was obtained for three sample solutions of different concentrations (0.10, 0.20, and 0.30 mg · mL^−1^) by an analyst who performed the analysis over a period of two weeks. To determine the precision of the procedure, the concentrations were prepared independently and experiments were performed in three different analyses. The precision was evaluated as the relative standard deviation (coefficient of variation, CV [%]).

#### 2.8.5. Detection Limit and Quantitative Limit Based on the Calibration Curve

A specific calibration curve was studied using samples containing acetaminophen in the range of the detection limit, namely, 0.125, 0.250, and 0.400 *μ*g · spot^−1^. The experiments were performed in six different analyses.

The detection limit (DL) was calculated as

(1) DL=3.3σS.

The quantitative limit (QL) was calculated as

(2) QL=10σS,

where *σ* is the standard deviation of the response and *S* is the slope of the calibration curve.

#### 2.8.6. Robustness

The robustness of the method was tested according to guidelines described in the papers by Nagy-Turák and Ferenczi-Fodor et al. [34, 35, 37]. The robustness of the method was checked by spotting sample solution (1.00 *μ*g · spot^−1^) on the plate and developing the plate after altering the conditions (Table 1). The conditions changed were the sorbent type, the chamber type, extraction time, the temperature of plate activation, the distance of development, the wavelength, and the analyst. The method conditions and the selected factors with the values of their (+) and (−) levels are summarized in Table 1. A high level is represented by “+” and a low level by “−”. The main effects of seven factors were tested on two levels in eight experiments [35, 37]. The levels of factors investigated and the experimental design matrix (2^3^) are shown in Tables 1 and 2, respectively. The ways of calculation of the effects (*E*) characterizing the particular individual factors and rank probabilities [38] were early presented [35, 37, 39–43].

### 2.9. Comparison with Pharmacopeial Method

When developing a new analytical method, it is desirable to compare the results from the new method with those from an accepted method. Sample solutions were analyzed by TLC-densitometric method and by the method recommended in the Polish Pharmacopeia, namely, the UV-spectrophotometric method [31]. The equivalent of 100 mg of acetaminophen with 15 mL of hydrochloric acid (3.6 g/L) was shaken for 30 minutes, and then sufficient hydrochloric acid (3.6 g/L) was added to produce 100 mL and filtered. The 1.0 mL of filtrate was diluted to 100.0 mL with hydrochloric acid (3.6 g/L), and the absorbance of the resulting solution was measured at the maximum of 245 nm using Specord 205 (Analytik Jena, Germany). The content of acetaminophen was calculated taking 668 as the value of absorbance (1%, 1 cm) at the maximum of 245 nm.

The comparison of TLC-densitometric method with UV-spectrophotometric method to determine acetaminophen in pharmaceutical preparations was studied by use of ten independently repeated different analyses.

## 3. Results and Discussion

### 3.1. Validation of TLC Method

**Figure 1:**
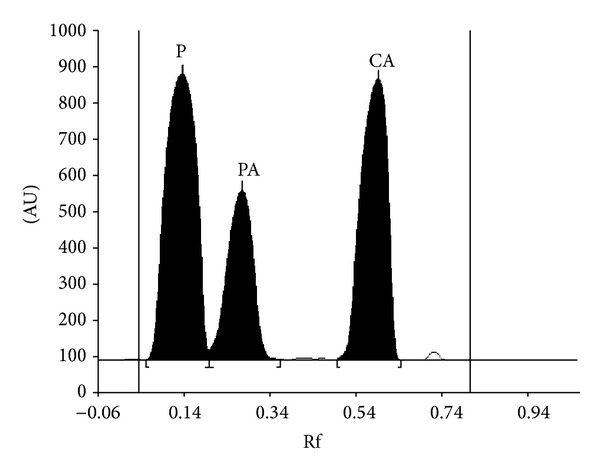
Densitogram obtained from acetaminophen standard (P) spiked with related substances, namely, p-aminophenol (PA) and 4′-chloroacetanilide (CA).

**Figure 2:**
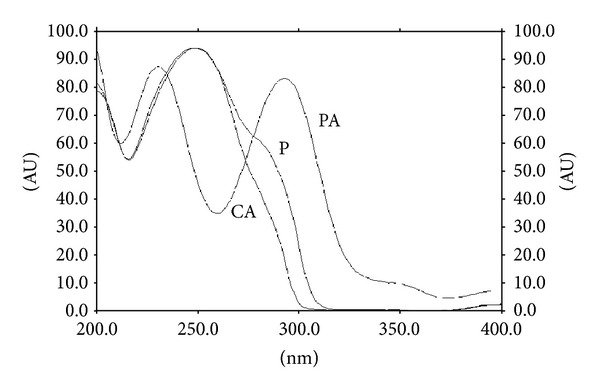
Spectrodensitograms of acetaminophen standard (P), p-aminophenol (PA), and 4′-chloroacetanilide (CA).

**Figure 3:**
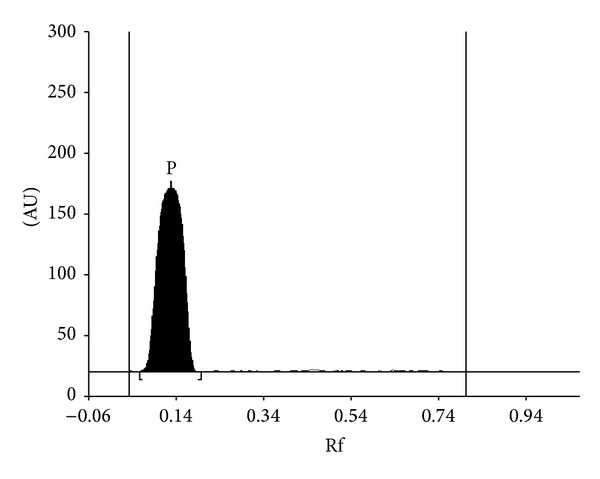
Densitogram of acetaminophen (P) coming from *Product 2* sample.

**Figure 4:**
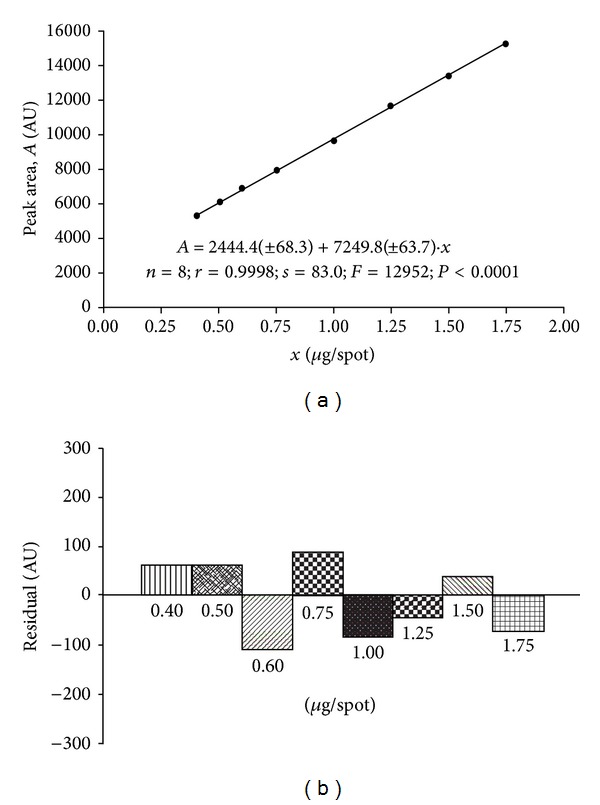
Calibration plot (a) and plot of residuals (b) for acetaminophen in the linear working range.

Summarized results of the method of validation are presented in Figures 1, 2, 3, and 4 as well as in Tables 1, 2, and 3. 

#### 3.1.1. Specificity

As was reported in Introduction part, our literature survey shows that many papers described the determination of acetaminophen in commercial products with the use of TLC and TLC-densitometry [24–30]. Various chromatographic conditions such as mobile phase composition and the kind of TLC plates were studied to obtain satisfactory results of acetaminophen qualitative and quantitative analysis. Based on the literature reports, our TLC-densitometry of acetaminophen was performed on aluminium plates precoated with silica gel 60F_254_ (recommended in literature for acetaminophen TLC analysis). In order to choose the optimal mobile phase which allows separating acetaminophen and its related substances (4-aminophenol and 4′-chloroacetanilide) from commercial product, different compositions and ratios of ammonia, toluene, ethyl acetate, chloroform, cyclohexane, methanol, n-hexane, acetone, and glacial acetic acid were examined. Of all mobile phases used in experiment, the mixture of chloroform + acetone + ammonia (25%) in volume compositions 8 : 2 : 0.1 as mobile phase resulted in compact bands and sharp and symmetric peaks of acetaminophen (P), related substances, namely, p-aminophenol (PA) and 4′-chloroacetanilide (CA). The mixture of chloroform + acetone + ammonia (25%) (8 : 2 : 0.1, v/v/v) used as mobile phase resulted in optimum migration acetaminophen (*R*_*F*(P)_ = 0.14 ± 0.01) and resolution of the drug from its related substances, namely, p-aminophenol (*R*_*F*(PA)_ = 0.28 ± 0.02) and 4′-chloroacetanilide (*R*_*F*(CA)_ = 0.59 ± 0.02) without interference from other components of the formulations matrix. When excess p-aminophenol and 4′-chloroacetanilide were added to the sample solution to check the specificity of the method the chromatogram presented the resolution of the acetaminophen peak from the related substance (p-aminophenol, and 4′-chloroacetanilide) peaks were equal to *R*_*S*(P/PA)_ = 1.25, *R*_*S*(PA/CA)_ = 2.76, *R*_*S*(P/CA)_ = 3.73 (Figure 1). Spectrodensitograms of acetaminophen (P), p-aminophenol (PA), and 4′-chloroacetanilide (CA) are presented in Figure 2. The absorption maximum of acetaminophen, p-aminophenol, and 4′-chloroacetanilide are equal to 248, 200, and 249 nm, respectively. Typical densitogram of acetaminophen coming from the extract of *Product 2* is presented in Figure 3. The identical densitograms were also obtained for the extract of *Products 1 and 3*. It was observed that excipients present in the formulation did not interfere with the acetaminophen peak. It was also stated that the analyte was not decomposed during development of the chromatogram and was stabled in solution and on the sorbent at room temperature. These facts indicate that new method is specificity.

#### 3.1.2. Accuracy

The accuracy of the method was evaluated by measurement of recovery (Table 3). When known amounts of acetaminophen were added to powdered tablets of known acetaminophen, content quantitative recoveries of 99.27% ÷ 102.34% (mean 100.58%) were obtained (Table 3). The low coefficient of variation values (CV < 2%) are indicative of the accuracy of the method.

#### 3.1.3. Calibration and Range

The statistical data shown in Table 3 and Figure 4(a) indicate that linear relationship exists between area of peaks [AU] and concentration of acetaminophen standard [*μ*g · spot^−1^]. The plot (*n* = 8) was linear in the range 0.40 to 1.75 *μ*g · spot^−1^ for NP-TLC analysis. The graphs of residuals against the concentration of acetaminophen were also plotted (Figure 4(b)). It was observed that the residuals were distributed both above and below the zero residuals line.

#### 3.1.4. Precision

The precision of the method was studied as repeatability and intermediate of the system at three different concentrations of tablet extractions. The results from these experiments, expressed as the coefficients of variation (CV, %) of the, respectively, response factors (a relationship between the peak area and concentration of acetaminophen) are presented in Table 3. Because CV for repeatability and intermediate were <3%, the method was precise.

#### 3.1.5. Detection Limit (DL) and Quantitative Limit (QL) Based on the Calibration Curve

The limits of detection and the limit of quantification were 0.09 *μ*g · spot^−1^ and 0.27 *μ*g · spot^−1^, respectively.

#### 3.1.6. Robustness

**Figure 5:**
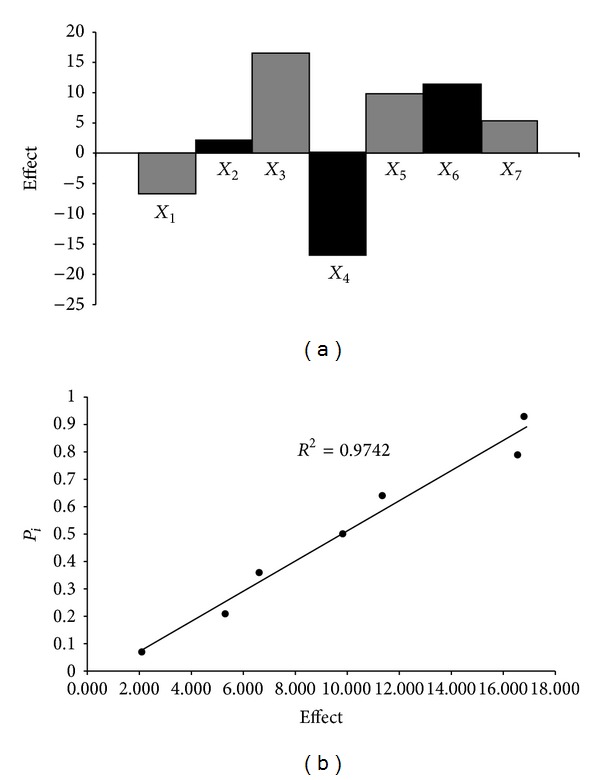
Robustness test: the effects of factors (a) and half-normal probability plot of effects (b) for determination of acetaminophen in *Product 2*.

The main effects of seven factors were tested on two levels in eight experiments (Tables 1 and 2) for determination of robustness applied NP-TLC method.  Table 2 shows the results obtained for acetaminophen content (*y*_*i*_) in three pharmaceutical preparations coded: *P1 (Product 1), P2 (Product 2), and P3 (Product 3)*. The main effects of the factors calculated from these results (*y*_*i*_) are also presented in Table 2. These results show that no factor has significant effect on the results. These results were also evaluated by half-normal probability plotting of rank probabilities (*p*_*i*_) as a function of the absolute values of the main effects [35, 38, 39]. For example, the effects of factors and half-normal probability plot of effects for the determination of acetaminophen in *Product 2* are presented in Figure 5. Similarly, results were obtained for determination of acetaminophen in *Product 1* and *Product 3*. The points of all factors lie near the straight line, which indicates that their effect is negligible. Based on the results of the robustness test (Table 2, experimental design matrix (2^3^) for robustness), which were determined according to the guidelines described in the papers by Nagy-Turák and Ferenczi-Fodor et al., it was stated that the proposed TLC-densitometric method can be regarded as robust. Moreover, it can be suggested that the modification of robustness test procedure in comparison to the methodology presented by Nagy-Turák and Ferenczi-Fodor et al. [35, 37] and to our previous investigations [39–43] is suitable for robustness study of TLC-densitometric method used in acetaminophen determination in combined pharmaceutical dosage form. 

It was stated that besides the robustness factors presented in our previous validation studies concerning TLC-densitometric investigations of different bioactive substances in their pharmaceutical preparations like hydrocortisone, naproxen, tocopherol acetate, and acetylsalicylic acid (e.g., volume of n-hexane, saturation time of the chamber, etc.), the changes of the following chromatographic condition such as sorbent type, the chamber type, extraction time, the temperature of plate activation, the distance of development, the wavelength, and the analyst used in this work can be successfully applied as the new factors in robustness test of TLC-densitometric method which is successfully applied in drug analysis. 

#### 3.1.7. Analysis of Acetaminophen in Commercial Tablets

A single spot at an average *R*_*F*_ equal 0.14 was observed in the chromatograms obtained from the extract of acetaminophen tablets. There was no interference from excipients present in the tablets. It was also concluded that no degradation of acetaminophen had occurred in the formulation analyzed by the TLC method. In each case, the *R*_*F*_ values of acetaminophen standard and acetaminophen from *Product 1, Product 2, and Product 3* were equal to 0.14 ÷ 0.01 for TLC-densitometric analysis. The identities of acetaminophen standard with acetaminophen from the commercial samples were investigated on the basis of the comparison of their spectra. The very good correspondence between spectrodensitograms was stated. In all cases the absorption maximum (*λ*_max⁡_) is equal to 248 nm. The purities of acetaminophen peaks from the samples of *Product 1, Product 2, and Product 3* were also assessed by comparing the spectra obtained from a acetaminophen standard at the peak start, peak apex, and peak end of spot. It was found that *r*(*S*, *M*) > 0.999 and *r*(*M*, *E*) > 0.999 for all of the analyses performed by the TLC-densitometric technique. Statistical data concerning the results of quantitative determination of acetaminophen in the ten repeated different analyses of pharmaceutical preparations are presented in Table 4. It was stated that acetaminophen amounts in pharmaceutical preparations determined by TLC-densitometric method are equal to 99.3%, 99.8%, and 100.8%, respectively, for *Product 1, Product 2, and Product 3* in relation to the amounts of acetaminophen declared by the manufacturers. 

### 3.2. Comparison with Pharmacopeial Method

To verify the results obtained by the TLC-densitometric method, comparison was made with a previous report using the Polish Pharmacopeia UV-spectrophotometric method [31]. When compared with the pharmacopeial method recommended for acetaminophen tablets, similar results were obtained for ten repeated different analyses (Table 4). The average assays of acetaminophen were 496.7 ± 6.4 mg · tablet^−1^ and 495.7 ± 7.0 mg · tablet^−1^ *Product 1*, 499.1 ± 5.6 mg · tablet^−1^ and 497.5 ± 4.8 mg · tablet^−1^ *Product 2*, 503.8 ± 5.1 mg · tablet^−1^ and 501.4 ± 7.9 mg · tablet^−1^ *Product 3* for TLC-densitometric and UV-spectrophotometric methods, respectively. The coefficients of variance were smaller than 2% in each case. High reproducibility and insignificant differences between the two compared methods were obtained at the 95% probability level for *t*-test and *F*-test of significance of 0.34 < 2.10 and 1.24 < 3.18; 0.69 < 2.10 and 1.36 < 3.18; 0.81 < 2.10 and 2.39 < 3.18; respectively, for *Product 1*, *Product 2, and Product 3*. These results confirmed statistically that TLC-densitometric method is accurate and can be used as a substitute method. 

Acetaminophen content in investigated pharmaceutical preparations is consistent with that reported by the Polish Pharmacopoeia [31]; acetaminophen content in the preparation should not be smaller than 95% and larger than the 105% of the declared value.

The results of this study and those presented in our previous papers [39–43] indicate that the validated TLC-densitometric method can be successfully applied to the determination of biological active compounds in selected pharmaceutical formulations.

## 4. Conclusion

Our TLC-densitometric study of acetaminophen indicates that of all applied chromatographic conditions, the most suitable are aluminium plates precoated with silica gel 60F_254_ as stationary phase and a mixture of chloroform + acetone + ammonia (25%) in volume compositions 8 : 2 : 0.1 as mobile phase. Above-mentioned chromatographic conditions resulted in optimum migration of acetaminophen and complete resolution of this compound from its related substances, namely, 4-aminophenol and 4′-chloroacetanilide from commercial products in combined dosage form. This fact confirmed the specificity of proposed method. According to the results of the experiments performed using the TLC-densitometric method, it was determined that the procedure used in this study is reliable with specificity, accuracy, precision, and robustness. The TLC-densitometric method also realizes the criterion of the linearity in the required range of acetaminophen concentrations. The results of validation of developed TLC-densitometric method for acetaminophen analysis indicate that the changes of the following chromatographic conditions such as sorbent type, the chamber type, extraction time, the temperature of plate activation, the distance of development, the wavelength, and the analyst used in our work can be successfully used as the new factors in robustness test of TLC-densitometric method, which is widely applied in drug analysis. 

Comparison of the acetaminophen content in tablets obtained from the TLC-densitometry to those determined by the use of the official UV-spectrophotometric method shows that acetaminophen content in investigated pharmaceutical preparations is consistent with that reported by the Polish Pharmacopoeia (is not smaller than 95% and not larger than the 105% of the declared value). It could be said that the TLC-densitometric and UV-spectrophotometric methods mentioned in this study are suitable for the routine analysis of acetaminophen in quantity control laboratories. Moreover, the results in our work confirmed statistically that TLC-densitometric method is accurate and can be used as a substitute method for the accurate assay of the acetaminophen in pharmaceutical dosage forms, for example, in situation when UV-spectrophotometer or HPLC-UV is not affordable in laboratory.

## Figures and Tables

**Table 1:** The factors and their levels investigated in robustness test.

Symbol	Factors	Method condition	Levels	
			+	−
*X*_1_	Sorbent type (Merck, #)	Al sheet (1.05554)	Al sheet (1.05554)	Al sheet (1.05570)
*X*_2_	Chamber type	Twin trough, 20 cm × 20 cm	Twin trough, 20 cm × 20 cm	Twin trough, 20 cm × 10 cm
*X*_3_	Temperature of plate activation (°C)	120	130	110
*X*_4_	Extraction time (min)	5	6	4
*X*_5_	Distance of development (cm)	7.5	8.0	7.0
*X*_6_	Wavelength (nm)	248	250	246
*X*_7_	Analyst	A	A	B

**Table 2:** Experimental design matrix (2^3^) for robustness test.

Experiment no.	*X*_1_	*X*_2_	*X*_3_	*X*_4_	*X*_5_	*X*_6_	*X*_7_	Acetaminophen content (*y*_*i*_) [mg · tablet^−1^] in tablets^a^		
								P1	P2	P3
1	+	+	+	+	+	+	+	499.0	494.5	501.2
2	+	+	−	+	−	−	−	454.2	451.6	459.8
3	+	−	+	−	−	+	−	496.4	494.1	496.7
4	+	−	−	−	+	−	+	485.1	481.4	488.2
5	−	+	+	−	+	−	−	501.1	501.3	503.4
6	−	+	−	−	−	+	+	495.8	491.5	492.5
7	−	−	+	+	−	−	+	478.0	477.9	485.6
8	−	−	−	+	+	+	−	485.9	477.2	483.5

Effect of the drug										
P1	−6.525	1.175	13.375	−15.325	11.675	14.675	5.075			
P2	−6.579	2.078	16.521	−16.777	9.804	11.297	5.278			
P3	−4.775	0.725	15.725	−12.675	10.425	9.225	6.025			

^a^ *Product* 1 (P1), *Product* 2 (P2), *Product* 3 (P3).

**Table 3:** Method-validation data for the quantitative determination of acetaminophen by NP-TLC with densitometry^a^.

Method characteristic	Results		
Specificity	Specific		
Range [*μ*g · spot^−1^]	0.40 ÷ 1.75		
Linearity [*μ*g · spot^−1^]	*A* = 2444.4(±68.3) + 7249.8(±63.7) · *x *		
	*n* = 8; *r* = 0.9998; *s* = 83.0; *F* = 12952; *P* < 0.0001		
Detection limit (DL) [*μ*g · spot^−1^]	0.09		
Quantitation limit (QL) [*μ*g · spot^−1^]	0.27		

	For tablets		
	*Product* 1	*Product* 2	*Product* 3

Accuracy			
For 50% acetaminophen added (*n* = 6)	*R* = 100.72%; CV = 1.13%	*R* = 99.69%; CV = 0.91%	*R* = 101.34%; CV = 1.29%
For 100% acetaminophen added (*n* = 6)	*R* = 100.58%; CV = 1.21%	*R* = 99.63%; CV = 0.92%	*R* = 102.34%; CV = 1.33%
For 150% acetaminophen added (*n* = 6)	*R* = 101.23%; CV = 0.86%	*R* = 99.27%; CV = 1.12%	*R* = 100.87%; CV = 1.08%

Precision (CV, [%])			
*Repeatability *			
For 0.50 *μ*g · spot^−1^ (*n* = 3)	0.57	0.85	0.99
For 1.00 *μ*g · spot^−1^ (*n* = 3)	0.66	0.76	0.78
For 1.50 *μ*g · spot^−1^ (*n* = 3)	1.00	0.73	1.11
*Intermediate *			
For 0.50 *μ*g · spot^−1^ (*n* = 3)	0.85	1.41	1.11
For 1.00 *μ*g · spot^−1^ (*n* = 3)	1.20	1.57	0.99
For 1.50 *μ*g · spot^−1^ (*n* = 3)	1.13	1.36	1.56

Robustness (CV, [%])	Robust	Robust	Robust

^a^ *A*: peak area [AU], *x*: amount [*μ*g · spot^−1^] of drug analyzed, *r*: correlation coefficient, *R*: recovery [%], CV: coefficient of variation [%].

**Table 4:** Acetaminophen assay [mg · tablet^−1^] obtained from ten repeated different analyses by TLC-densitometric and UV-spectrodensitometric methods for tablets of three different pharmaceutical manufactures.

No.	*Product* 1		*Product* 2		*Product* 3	
	Assay by TLC-densitometric method	Assay by UV-spectrophotometric method	Assay by TLC-densitometric method	Assay by UV-spectrophotometric method	Assay by TLC-densitometric method	Assay by UV-spectrophotometric method
1	500.3	502.3	509.0	504.5	502.4	505.7
2	503.5	491.3	488.1	497.5	496.8	489.8
3	488.3	486.5	499.6	494.7	509.6	511.2
4	495.2	494.4	504.4	498.6	497.8	492.3
5	500.5	493.5	494.3	503.6	502.7	508.9
6	491.8	490.6	499.6	489.9	505.8	506.7
7	489.1	494.5	499.2	497.3	497.6	491.2
8	504.2	506.2	501.5	501.9	509.8	505.9
9	503.1	506.9	496.9	492.9	506.7	497.9
10	491.2	490.8	498.8	493.9	508.9	504.6
Average assay	496.7	495.7	499.1	497.5	503.8	501.4
Label claimed	500.0	500.0	500.0	500.0	500.0	500.0
Standard deviation (SD)	6.4	7.0	5.6	4.8	5.1	7.9
Coefficient of variation [CV, %]	1.27	1.40	1.12	0.96	1.01	1.58

TLC-densitometric method compared with UV-spectrophotometric method						
*t*-test						
*t* calculated	0.34		0.69		0.81	
*t*_(95%,18)_ tabulated	2.10		2.10		2.10	
*F*-test						
*F* calculated	1.24		1.36		2.39	
*F*_(95%,*f*1=*f*2=9)_ tabulated	3.18		3.18		3.18	
